# Study on the regulation mechanism of TBX5 gene and Gegen Qinlian decoction on colorectal cancer

**DOI:** 10.3389/fonc.2025.1732015

**Published:** 2026-01-14

**Authors:** Dong Chen, Yan Cai, Wencang Gao, Dexiang Pang, Senquan Yu

**Affiliations:** 1 The Second Affiliated Hospital of Zhejiang Chinese Medical University (Xinhua Hospital of Zhejiang Province), Hangzhou, China; 2 School of Basic Medical Sciences, Zhejiang Chinese Medical University, Hangzhou, China; 3 Key Labortary of Blood-stasis-toxin Syndrome of Zhejiang Province, Hangzhou, China; 4 Traditional Chinese Medicine’Preventing Disease’ Wisdom Health Project Research Center of Zhejiang, Hangzhou, China

**Keywords:** colorectal cancer, Gegen Qinlian decoction, molecular docking, networks, Tbx5

## Abstract

**Background:**

TBX5 is a key protein regulated by the TGF-β/Smad3 pathway, which plays a significant role in colorectal cancer (CRC) development. This study aims to investigate the interplay between Gegen Qinlian Decoction (GQD), TBX5, and CRC mechanisms.

**Methods:**

The Cancer Genome Atlas (TCGA-CRC) dataset from the TCGA database was utilized for the study. Differential expression analysis was conducted to identify differentially expressed genes (DEGs) and differentially expressed miRNAs (DEMs). Subsequently, analyses were performed focusing on TBX5, encompassing pan-cancer evaluation, protein-level expression assessment, and enrichment analysis. Correlation analyses were carried out to investigate the relationships between TBX5, clinical characteristics, prognosis, and key pathways in CRC. Furthermore, immune analysis, network construction, drug prediction, and molecular docking studies were conducted.

**Results:**

A total of 1,834 differentially expressed genes (DEGs) were identified in TCGA-CRC. TBX5 exhibited elevated expression levels in CRC compared to other cancer types and normal tissues, correlating with lower survival rates in the high TBX5 expression cohort. Additionally, SPTBN4, RP11-35N6.1, CHD5, SLC4A8, KLHL32, MAP2, and ATP8A2 were found to be prognostic markers for patient outcomes. Age, N, and M stages were identified as significant independent prognostic factors for CRC. Notably, there was a significant upregulation of cell stemness, epithelial-mesenchymal transition (EMT), and angiogenesis-related genes in the high TBX5 expression group. Furthermore, the prognostic significance of 10 immune cell types, including mast cells, myeloid-derived suppressor cells (MDSCs), and monocytes, was evident. Thirteen key miRNAs were identified, and an mRNA-miRNA-lncRNA regulatory network was constructed, revealing relationships such as TBX5-hsa-mir-1270-TMPO-AS1. Molecular docking analyses demonstrated favorable binding of active compounds like formononetin and cinnamic acid to TBX5.

**Conclusion:**

Our findings suggested a potential association between GQD, TBX5-related genes, and CRC progression, which could provide valuable insights for the diagnosis and treatment of CRC.

## 1 Introduction

Colorectal cancer (CRC) is a leading cause of global mortality and morbidity, ranking second in cancer-related deaths (1). In 2020, there were an estimated 1,148,515 new cases of CRC worldwide, making it the fifth most commonly diagnosed cancer, with a rising incidence among younger individuals (2). The etiology of CRC is complex, involving environmental factors (such as diet, tobacco, and alcohol), interactions with pathogenic microflora, genetic predisposition, and immune responses (3–8). Treatment options for CRC include surgery, radiotherapy, and chemotherapy, but not all patients respond well to these therapies. Early detection of CRC is challenging due to its subtle symptoms, leading to a minority of cases being diagnosed at early stages. Patients with CRC and distant metastases have a poor prognosis, with only a 14% 5-year survival rate (9). Consequently, there is a growing focus on understanding the biological mechanisms, improving diagnostic methods, developing new treatments, and identifying prognostic markers for CRC.

The T-box transcription factor gene (TBX) encodes evolutionarily conserved transcription factors crucial for organogenesis and development. Alternative splicing generates distinct TBX subtypes with roles in cell proliferation (10). Mutations in TBX gene family members can lead to developmental syndromes and are implicated in certain cancers (11). TBX5, located on human chromosome 12q24.1, consists of 8 exons encoding a 518-amino acid protein. It is expressed in various tissues, with highest levels in the heart and lung (12). TBX5 binds to the T-box element of target genes, regulating heart differentiation. Recent studies have explored TBX gene expression in normal and cancer tissues, suggesting its potential as a biomarker in lung cancer metastasis (13, 14). TBX5 influences pulmonary branch morphogenesis through the fibroblast growth factor 10 (FGF10) signaling pathway (13). Overexpression of TBX5 inhibits colony formation, induces apoptosis, and suppresses cell growth. Its expression hinders development in normal cells and proliferation and migration in cancer cells (15, 16). Further research is needed to investigate TBX5 in colorectal cancer, particularly its tumor suppression mechanisms for diagnosis and prognosis.

Gegen Qinlian decoction, originating from the Treatise on Febrile Diseases, comprises Radix Puerariae, Scutellaria baicalensis, Coptis chinensis, and licorice. This ancient formula, attributed to the renowned physician Zhang Zhongjing, aims to address the syndrome of unresolved exterior and interior heat invasion. With a history of more than two millennia, its therapeutic effects encompass exterior and interior dredging, alleviating symptoms such as fever, dry mouth, sweating, red or yellow tongue, rapid pulse, and intermittent stagnation. Contemporary pharmacological investigations have elucidated that baicalein, a compound present in Scutellaria baicalensis, can impede the synthesis and release of inflammatory mediators, thereby manifesting anti-inflammatory properties. Furthermore, the saponins in Radix Astragali exhibit anti-inflammatory and antibacterial characteristics, contributing to the mitigation of inflammatory responses (17). Recent research has indicated that the Gegen Qinlian decoction can potentiate the efficacy of PD-1 in inhibiting microsatellite instability in colorectal cancer by modulating the intestinal microbiota and tumor microenvironment (18).

Therefore, we employed bioinformatics analysis to investigate TBX5 gene expression in colorectal cancer (CRC), its correlation with clinicopathological features, and survival outcomes. Our study included immune infiltration analysis, drug prediction, network construction, and molecular docking to explore the therapeutic and regulatory effects of Gegen Qinlian decoction and TBX5 in CRC.

## 2 Materials and methods

### 2.1 Data source

Transcriptomic data from 638 colorectal cancer (CRC) tumour tissue (colon adenocarcinoma, COAD and rectum adenocarcinoma, READ) and 51 normal tissue samples of TCGA-CRC was acquired from the TCGA (http://cancergenome.nih.gov/) database. Normal tissue samples from 4,010 healthy donors were obtained from the GTEx database (https://gtexportal.org). All data were standardised using the FPKM method to eliminate the influence of gene length on expression levels and to correct for sequencing depth-induced biases across samples.

### 2.2 Difference analysis

Differentially expressed genes (DEGs) were obtained based on TCGA-CRC and differentially expressed miRNAs (DEMs) were obtained based on TCGA-CRC miRNA by differential analysis (p.adj < 0.05, |log2FC|>1) between CRC and normal groups using the DESeq2 package (19).

### 2.3 Pan-cancer and protein level expression analyses

To investigate the expression of TBX5 in several cancers, TBX5 expression was analysed in 33 tumour and normal tissues using the TCGA and Genotype Tissue Expression (GTEx) databases. Protein expression of TBX5 was analysed between CRC and normal tissues using the Human Protein Abstracts Database (HPA, https://www.proteinatlas.org/).

### 2.4 Correlation analysis of prognosis, independent prognostic factors and key pathway

Based on TBX5 expression, CRC patients were categorised into high and low expression groups. To analyse the relationship between TBX5 and the prognosis of CRC patients, Kaplan-meier (K-M) survival analysis was executed. To analyse the relationship between TBX5-related genes and the prognosis of CRC patients, spearman analysis was executed between TBX5 and DEGs. Based on the expression of the TBX5-related genes, patients were classified into high and low expression groups using the survminer package to calculate the optimal cutoff value, and K-M survival analysis was executed.

TBX5 and clinical characteristics (age, gender, T, N, and M stages) were selected to build a nomogram for predicting CRC patient survival. Firstly, based on the samples with survival information in TCGA-CRC, univariate Cox regression analysis was performed using the coxph function from the “survival” package (v 3.5-3) (20) [with p<0.05, hazard ratio (HR)≠1]. Then, to ensure the validity and reliability of the univariate results, proportional hazards (PH) assumption test was conducted using the cox.zph function from the “survival” package (v 3.5-3) (with p>0.05). Subsequently, the “survival” package (v 3.5-3) was further used to perform multivariate Cox regression models on the selected variables (with p<0.05, HR≠1), and PH assumption test (with p>0.05) were conducted to identify the independent prognostic factors. The “forestplot” package (v 3.1.1) (21) was used to visually present the results of univariate and multivariate Cox analyses by creating forest plots. Then, to explore the predictive power of the independent prognostic factors on CRC patient survival rates, a nomogram for 1-, 3-, and 5-year survival rates of CRC patients was constructed using the “rms” package (v 6.5-1) (22). Finally, to verify the accuracy and reliability of the nomogram, calibration curves and ROC curves for 1-, 3-, and 5-year survival rates, as well as 1-, 3-, and 5-year overall survival periods, were respectively drawn using the “rms” package (v 6.5-1) and the “survivalROC” package (v 1.0.3) (23).

To validate the relationship of TBX5 with cell stemness, EMT pathway and angiogenesis related genes, single sample gene set enrichment analysis (ssGSEA) scores were calculated for each gene set using the ssGSEA algorithm of the GSVA package (24). Spearman analysis was executed between TBX5 gene expression and per scores.

### 2.5 Gene set enrichment analysis

GSEA was executed to explore the function of TBX5. Firstly, the c5.go.bp.v7.4.symbols.gmt, c5.go.cc.v7.4.symbols.gmt, c5.go.mf.v7.4.symbols.gmt and c2.cp.kegg.v7.4.symbols.gmt genes sets were obtained from the molecular signatures database (MsigDB) (http://www.broadinstitute.org/gsea/msigdb/) as a reference gene set. Then, Based on the TCGA-CRC dataset, differential analysis between all samples in two groups were executed and ranked from largest to smallest by multiples of variance. Finally, GO and KEGG enrichment analyses were executed using ClusterProfiler package (p.adjust<0.05) (25).

### 2.6 Immune infiltration analysis

To study the immune cell infiltration in the two groups, the ssGSEA algorithm was used to calculate the infiltration abundance of 28 immune cells between the two groups (26). The Wilcoxon signed-rank test was employed to compare differences in infiltration levels between the high- and low-expression groups of TBX5 within the training set. Concurrently, the Benjamini-Hochberg (BH) method was applied to correct the p-values, with both corrected and uncorrected results reported. Furthermore, to study the relationship between differential immune cells and CRC prognosis, K-M survival analysis was performed and significance of differences analysed using the log-rank test.

### 2.7 Construction of the network

To explore the function of TBX5, TBX5 was included in the GeneMANIA (http://www.genemania.org) database for analysis. The miRNAs associated with TBX5 were forecasted by targetScan databases (v 7.2, published March 2018). Taking intersections of miRNAs targeting TBX5 and DEMs to obtain key miRNAs. lncRNA associated with key miRNAs were predicted via STARBASE databases (v 2.0, published January 2014). Moreover, cytoscape software was applied to optimize the results of mRNA-miRNA-lncRNA network.

### 2.8 Drug sensitivity analysis and molecular docking

The 50% inhibitory concentration (IC50) of each of the 128 drugs in TCGA-CRC was calculated for each sample using pRRophetic package (27) (p.adjust<0.05). To investigate the regulatory relationship between Gegen Qinlian Decoction (GQD) and TBX5. The corresponding target protein for each active ingredient in GQD was predicted by TCMSP database. The five drug-like principles (Lipinski rule): relative molecular weight (MW) ≤ 500, hydrogen-bonded donor (Hdon) ≤ 5, hydrogen-bonded acceptor (Hacc) ≤ 10, lipid-water partition coefficient (AlogP) ≤ 5, the value of oral bioavailability (OB) ≥ 30% and vaule of drug-likeness (DL) ≥0.18 were used as the screening conditions. Target proteins were abstracted and converted to gene names using UniProtKB (http://www.uniprot.org). Spearman’s correlation analysis was employed to assess the relationship between TBX5 and drug target genes. For each drug, the single target gene exhibiting the strongest correlation with TBX5 was selected and subjected to molecular docking with TBX5. First, the three-dimensional structure of TBX5 was obtained from the Uniprot database (https://www.uniprot.org/), with the corresponding crystal structure PDB ID being 2X6U (https://doi.org/10.2210/pdb2X6U/pdb). The 3D structure of the GQD active ingredient was retrieved from the TCM Database@Taiwan (https://tcm.cmu.edu.tw/) and HERB 2.0 (http://herb.ac.cn). The CB-Dock online platform (http://cao.labshare.cn/cb-dock/) was employed to predict protein-compound binding affinity and binding sites. Subsequently, AutoDock Vina was utilised to perform precise molecular docking validation and visualisation of the predicted results (docking binding energy < -5 kcal/mol).

### 2.9 Culture of human colon cancer cells (HCT116)

The frozen cells were quickly thawed and dissolved. Subsequently, they were transferred to a centrifuge tube containing complete medium and centrifuged at 1000r/min to eliminate the supernatant. The cell suspension was then supplemented with DMEM complete medium, transferred to T-25 culture flasks, and incubated at 37°C with 5% CO_2_. Upon reaching 80% confluence, cell passaging was carried out. The cells underwent PBS rinsing, digestion with 0.25% trypsin for single-cell suspension generation, followed by centrifugation. Finally, the cells were combined with a cryopreservation solution and placed into cryovials. After freezing at -80°C, the cells were transferred to liquid nitrogen for prolonged storage.

### 2.10 Packaging of the lentivirus

The frozen Human embryonic kidney (293T) cells were immediately rewarmed at 37°C, put in complete medium containing 10 ml, and centrifuged at 1000 r/min for 5 min at room temperature. After removing the supernatant, the cells were put to DMEM complete media and transferred to T-25 culture flasks, where they were cultivated at 37°C with 5% CO_2_. After putting 293T cells in 4-5×10^6^/10cm dishes, incubate them for 24 hours at 37°C with 5% CO2. Plasmid No. 1 and the transfection reagent dilution No. 2 should be made in the following order **Supplementary Table S1**. After thoroughly combining No. 2 and No. 1 reagents, incubate the mixture at room temperature for 15 min. Drop by drop, 1 ml of the transfection mixture was added to the 293T cells when the cell density reached 80%-90%. Followed by, replace 10 ml of fresh 293T medium and virus medium 4–6 hours and 24 hours after transfection, respectively. Finally, cell culture supernatants were collected at 48, 72 hours post-transfection and centrifuged at 500 g for 10 min, after which the supernatants were collected for lentivirus infection.

### 2.11 Construction of stable cell lines

The cells were divided into sh-TBX5 group and sh-NC group for infection. First, wall-adherent cells were evenly distributed into 6-well plates at a density of 3 × 10^5 cells per well for 18–24 hours prior to lentiviral infection to ensure that there were approximately 6 × 10^5 cells in each well. After waiting for the cell-adherent fusion to reach 70%, the original medium was replaced with 2 ml of fresh medium containing 8 μg/ml polybrene and the appropriate amount of virus suspension was added. The incubation was continued at 37°C for 8 hours, and then the virus-containing medium was replaced with fresh medium. If the lentivirus contains fluorescent proteins, fluorescent expression can generally be observed 48 hours after transfection, and the fluorescent expression will be more obvious after 72 hours. If the results are not satisfactory, the infection operation can be repeated and continuous screening can be carried out by adding 2ug/ml puromycin to the medium until one month’s time.

### 2.12 Real-time quantitative-polymerase chain reaction

The total RNA was isolated with the help of TRIzol method. The SwsScript All-in-One First-strand-cDNA-synthesis SuperMIx for qPCR kit was used to synthesize cDNA. The SYBR Green qPCR Mix were used to perform the RT-qPCR reaction. Ct values were acquired, and the comparative gene expression was determined using the 2-ΔΔCt formula, with GAPDH serving as the internal control. Finally, GraphPad Prism 6 was used for data visualization and analysis. A list of primers was shown in **Supplementary Table S2**.

### 2.13 Animals

This study was approved by Institutional Animal Care and Use Committee of Zhejiang Center of Laboratory Animals and complied with the guidelines for animal care and use established by the Chinese government.15 female Balb/c nude mice were housed in individually ventilated cages, and the supplier ensured the high quality of the mouse strains through strict quality control measures. The study consisted of 3 groups of 5 mice each: the CRC group, the GQD group, and the TBX5 lentivirus transfection group.BALB/C-nu nude mice were inoculated with tumor cells in the right axilla.The nude mice were disinfected with an alcohol cotton ball wipe, fixed, and inoculated with 120 µL of cells in the right axillary subcutaneous area.The cotton swabs were pressed in the axilla after the completion of the inoculation to ensure that there was no leakage of cell suspensions.The growth status of the mice was observed on a daily basis, and the tumor was recorded once every 2 days. Tumor volume was recorded every 2 days.

### 2.14 Immunohistochemistry

Tumor tissue is taken and fixed in 4% paraformaldehyde solution, then dehydrated, embedded and sectioned. Sections are first antigenically repaired and blocked, then the TBX5 antibody is diluted 1:100 according to the antibody manufacturer’s instructions and the sections are incubated overnight in a 4°C refrigerator. The next day, sections are rewarmed in a 37°C warmer for 30 minutes, then goat anti-mouse/rabbit IgG secondary antibody is added and incubated at 37°C for 20 minutes. The final steps include DAB color development, hematoxylin re-staining, dehydration, transparency and sealing.

### 2.15 Statistical analysis

Statistical analysis was executed by R software. Differences between the two groups were executed by Wilcoxon test. The clusterProfiler was used to identified enrichment pathway.

## 3 Results

### 3.1 TBX5 expression was higher in CRC

**Figure 1:**
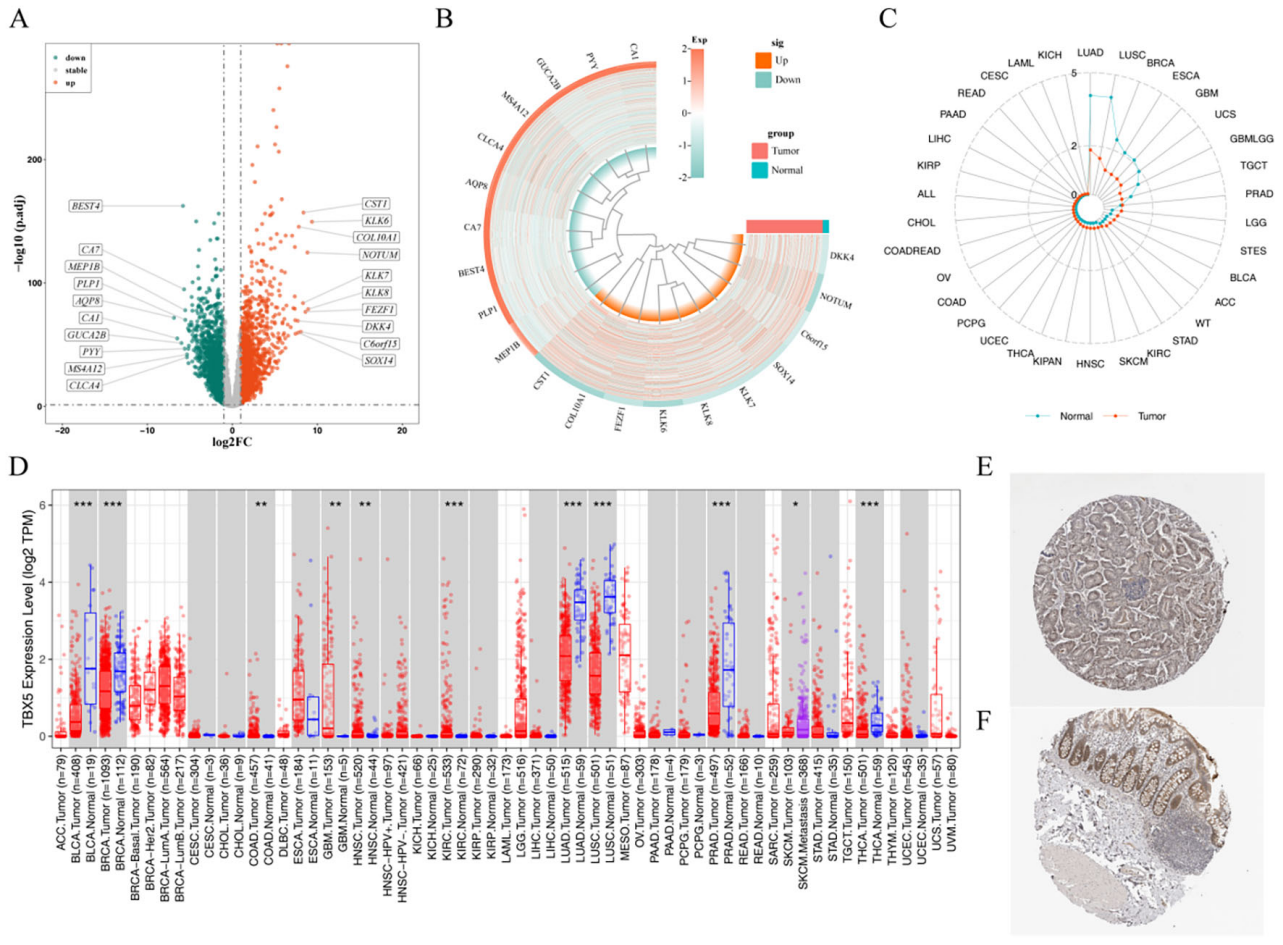
The transcription levels of TBX5 in human cancers. **(A)** Volcano map of differentially expressed genes in CRC. **(B)** Heat map of differentially expressed genes in CRC. **(C)** TBX5 expression in 33 tumour and normal tissues. **(D)** The mRNA expression of TBX5 between tumour and normal tissues was assessed using tissues from TCGA and GTEx database. Expression of TBX5 in CRC-COAD tissue **(E)** and normal tissue **(F)**. (.*p*-value ≤ 0.1; *p-value ≤ 0.05; **p-value ≤ 0.01; ***p-value ≤ 0.001; TBX5 gene symbol is TBX5).

A total of 3,681 DEGs (1,834 up regulated genes and 1,847 down regulated genes) were obtained by difference analysis (**Figures 1A, B**; **Supplementary Table S3**). Pan-cancer analysis showed that TBX5 expression was down-regulated in LUAD, LUSC, BRCA, ESCA, GBM, UCS, GBMLGG and TGCT, and up-regulated in other cancers, especially CRC-COAD (**Figures 1C, D**). Immunohistochemistry results showed that TBX5 was expressed at a higher level in CRC-COAD tissue compared to normal tissue. Moreover, in normal tissue, TBX5 was mostly expressed in the cytoplasm, whereas in CRC-COAD tissue it was mostly expressed in the nucleus, suggesting that the high expression of TBX5 into the nucleus played a regulatory role in CRC-COAD (**Figures 1E, F**).

### 3.2 SPTBN4, RP11-35N6.1, CHD5, SLC4A8, KLHL32, MAP2 and ATP8A2 were associated with CRC prognosis

**Figure 2:**
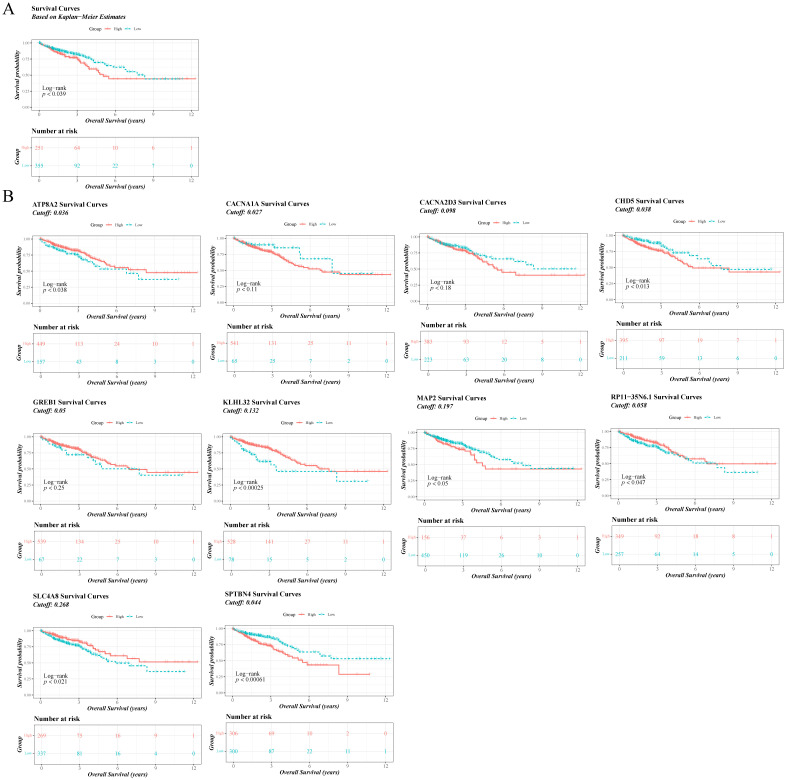
Differential analysis of different gene expressions associated with CRC prognosis. **(A)** Distribution of survival status of all samples of TBX5 in the high and low expression groups. **(B)** Distribution of survival status of all samples of CACNA1A, SPTBN4, RP11-35N6.1, CHD5, CREB1, SLC4A8, KLHL32, MAP2, CACNA2D3 and ATP8A2 in the high and low expression groups.

Patients were categorised into high and low expression groups based on TBX5 expression. K-M survival analysis showed that patients in the TBX5 high-expression group had a lower survival rate (**Figure 2A**). The absolute value of correlation coefficient top 10 genes including CACNA1A, SPTBN4, RP11-35N6.1, CHD5, GREB1, SLC4A8, KLHL32, MAP2, CACNA2D3, and ATP8A2 were obtained by spearman analysis. K-M results showed that PTBN4, RP11-35N6.1, CHD5, SLC4A8, KLHL32, MAP2, and ATP8A2 were obviously different between the two groups (**Figure 2B**).

**Figure 3:**
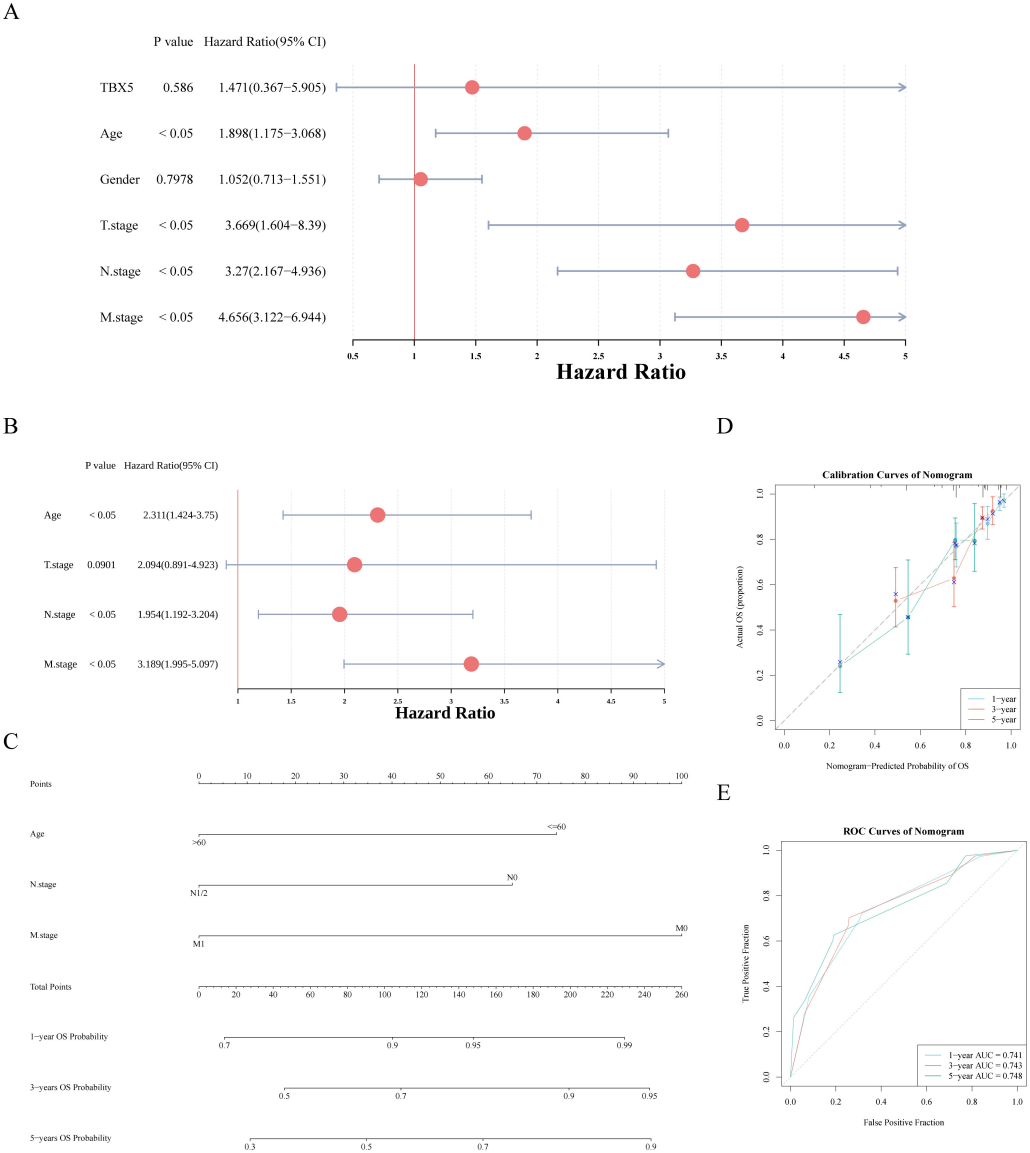
Construction of a nomogram. **(A)** Forest plot of univariate Cox analysis. **(B)** Forest plot of multivariate Cox analysis. **(C)** Nomogram construction. **(D)** Nomogram calibration curve. **(E)** Nomogram ROC curve.

The univariate Cox regression analysis forest plot (**Figure 3A**) and the PH assumption test (p>0.05) (**Supplementary Table S4-1**) showed that age, T, N, and M stages were significantly related to the survival of CRC patients (p<0.01). Furthermore, the multivariate Cox regression analysis forest plot (**Figure 3B**) and the PH assumption test (p>0.05) (**Supplementary Table S4-2**) indicated that age (HR = 2.311; 95%CI = 1.424-3.75), N (HR = 1.954; 95%CI = 1.192-3.204), and M stages (HR = 3.189; 95%CI = 1.995-5.097) remained significant independent prognostic factors (p<0.05). Additionally, a nomogram based on these independent prognostic factors was constructed as shown in **Figure 3C**. Moreover, the calibration curves demonstrated a high degree of overlap between the predicted probabilities of 1-, 3-, and 5-year survival from the nomogram and the reference line (**Figure 3D**). Also, the ROC curves of the nomogram model revealed that the AUC values at various time points were all above 0.7, indicating that the nomogram model had high a predictive value (**Figure 3E**). In summary, these findings are helpful for enhancing the predictive efficiency and therapeutic effect of CRC and improving patients’ prognosis.

### 3.3 Enrichment pathway of TBX5

**Figure 4:**
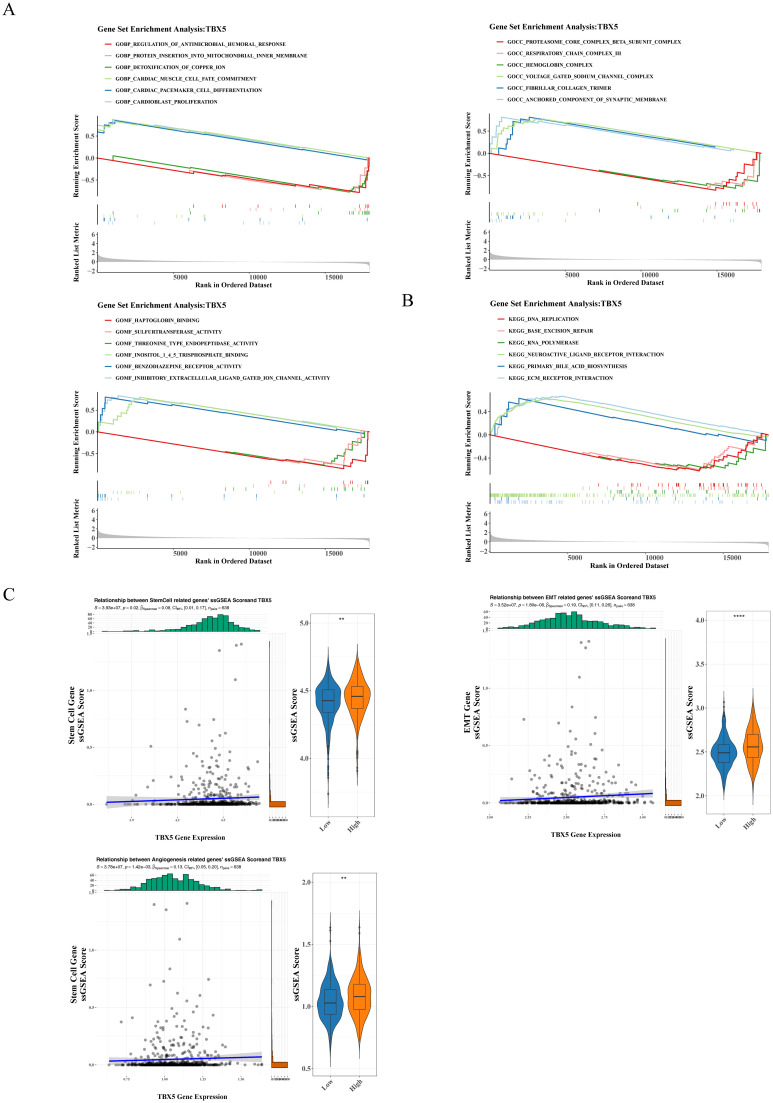
GO and KEGG enrichment analysis of TBX5. **(A)** GO enrichment analysis of TBX5 in BP **(a)**, CC **(b)**, and MF **(c)**. **(B)** KEGG GO enrichment analysis of TBX5. **(C)** Relationships between TBX5 and cell stemness, EMT and angiogenesis. (**p-value ≤ 0.01; ****p-value ≤ 0.0001; TBX5 gene symbol is TBX5).

Based on TBX5, GO and KEGG enrichment were executed. The results of GO displayed that TBX5 was enriched in biological processes (BP) such as cardioblast proliferation etc, cellular components (CC) such as anchored component of synaptic membrane etc, and molecular functions (MF) such as inhibitory extracellular ligand gated ion channel activity etc (**Figure 4A**; **Supplementary Table S5**). And TBX5 were enriched in ecm receptor interaction, etc KEGG pathway (**Figure 4B**; **Supplementary Table S6**). Correlation analysis showed significant correlation between TBX5 and cell stemness, EMT and angiogenesis but the correlation coefficient was low. The scores of cell stemness, EMT and angiogenesis-related genes were all obviously upregulated in the TBX5 high-expression group (**Figure 4C**).

### 3.4 A total of 10 differently expressed immune cells linked to patient prognosis

**Figure 5:**
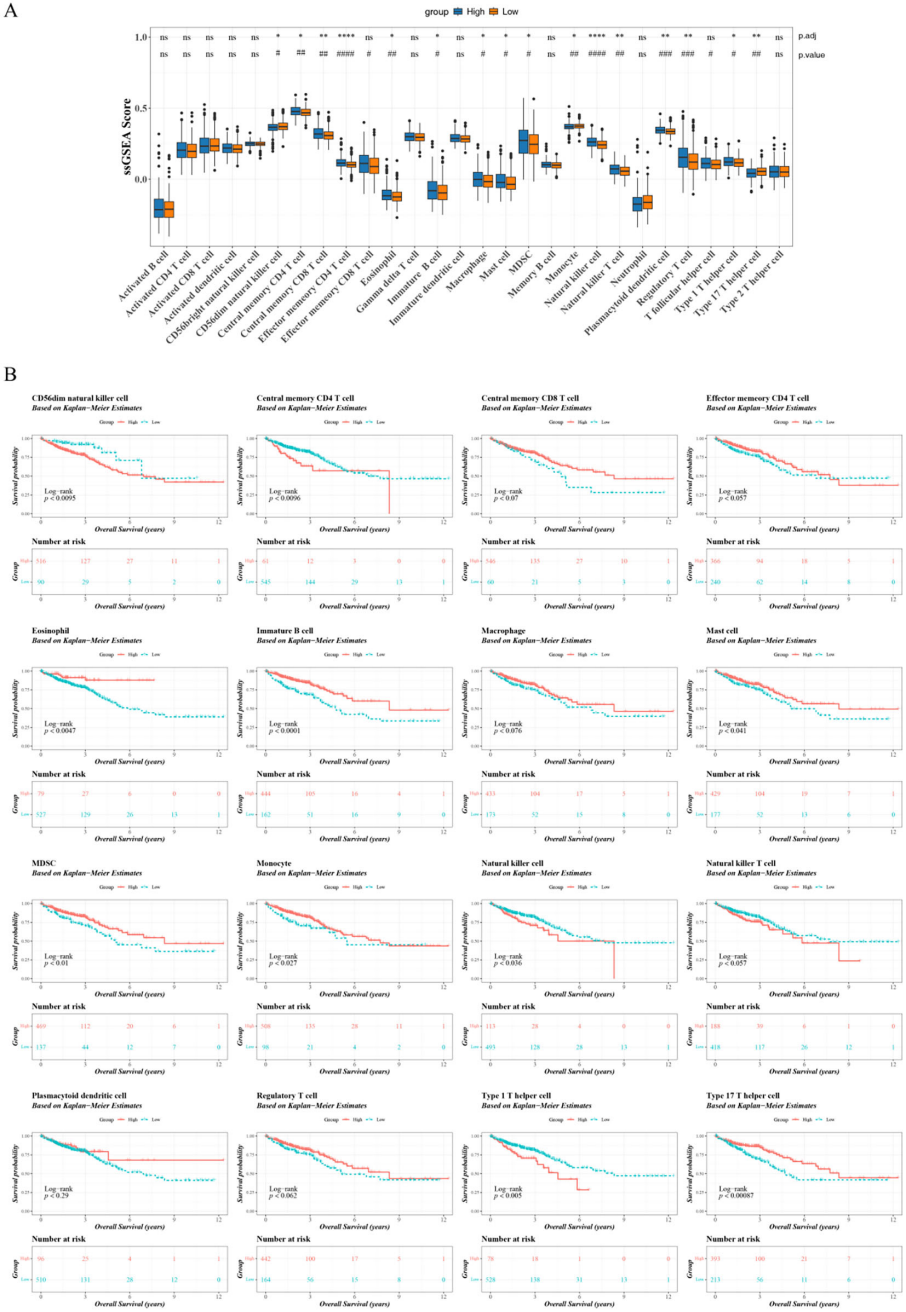
Differential analysis of differently expressed tumor-infiltrating immune cells linked to patient prognosis. **(A)** SsGSEA analysis to analyze the correlation between TBX5 expression and the immune cells linked to patient prognosis in the high and low expression groups. **(B)** Distribution of survival status of all samples of differential immune cells. (*p.adjust ≤ 0.05; **p.adjust ≤ 0.01; ***p.adjust ≤ 0.001; ****p.adjust ≤ 0.0001; #*p-value* ≤ 0.05; ##*p-value* ≤ 0.01; ###*p-value* ≤ 0.001; ####p.adjust ≤ 0.0001).

18 differently expressed immune cells (CD4 memory central T cells, monocyte, macrophage and mast cell etc) were obtained between the two groups. After BH correction, differences in these 16 immune cell types retained statistical significance. (**Figure 5A**). K-M results showed CD4 central memory T cells, eosinophil, B immature cells, mast cells, MDSC, monocyte etc were significantly associated with the prognosis of CRC patients (p value<0.05) (**Figure 5B**).

### 3.5 A total of 42 potential drugs were obtained

**Figure 6:**
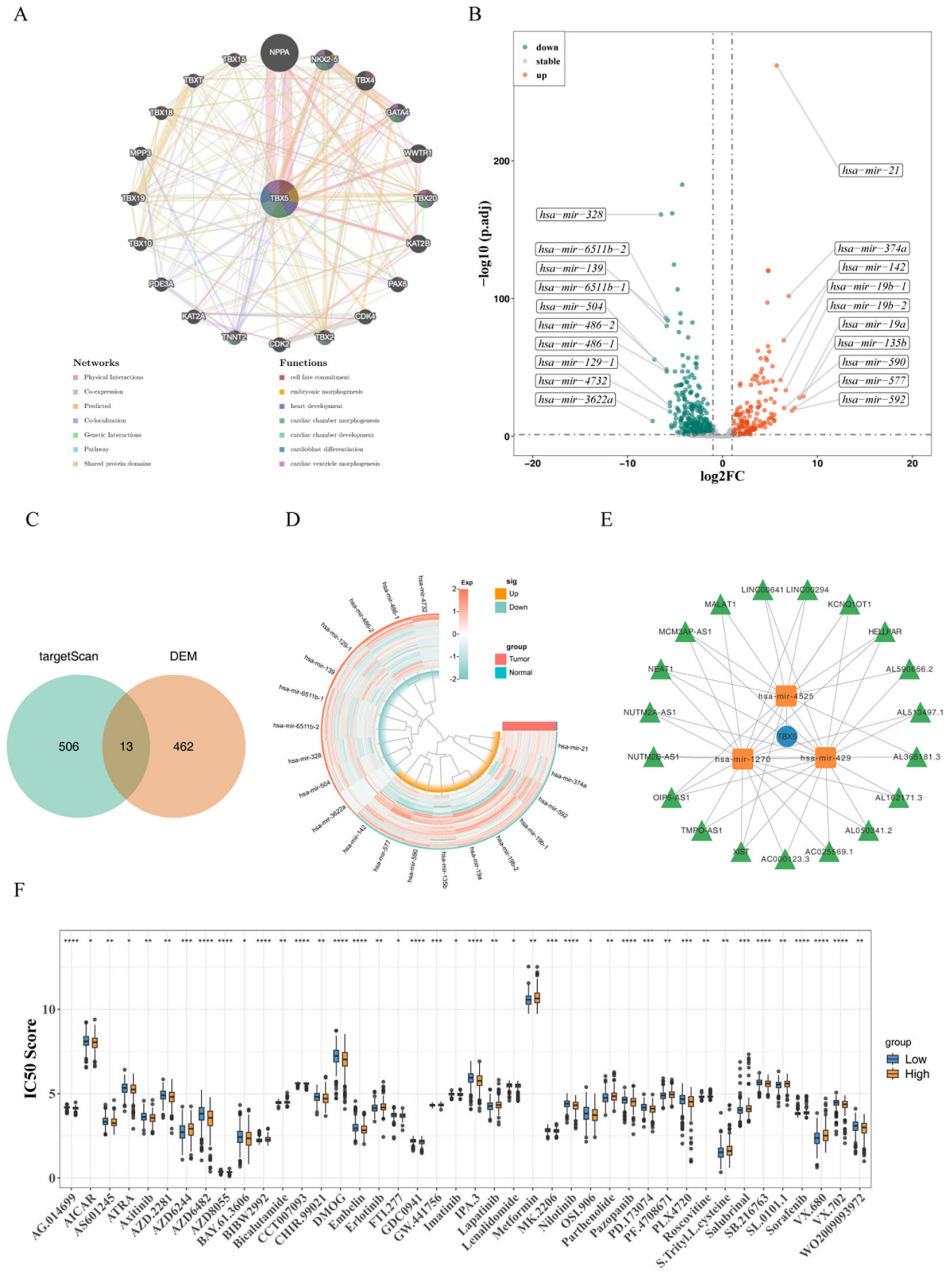
Differential analysis of differently expressed immune cells linked to patient prognosis. **(A)** Network selection of gene functions similar to TBX5 and associated pathways. **(B)** Volcano map of differentially expressed genes. **(C)** Heat map of differentially expressed genes. **(D)** Venn diagram of intersections of miRNAs targeting TBX5 and the genes. **(E)** Network of mRNA-miRNA-lncRNA targeting TBX5. **(F)** IC50 score analysis of high and low expression groups of TBX5 gene expression. (*p.adjust ≤ 0.05; **p.adjuste ≤ 0.01; ***p.adjust ≤ 0.001; ****p.adjust ≤ 0.0001; TBX5 gene symbol is TBX5).

A total of 21 genes functionally similar to TBX5 were obtained, including NPPA, TBX15 and NKX2–5 etc, and were mainly associated with the pathways of cell fate commitment, embryonic morpheogenesis, and heart development (**Figure 6A**). A total of 475 DEMs were obtained by difference analysis (**Figures 6B, C**; **Supplementary Table S7**). A total of 13 key miRNA were obtained by overlapping miRNAs targeting TBX5 and DEMs (**Figure 6D**).1 mRNA, 3 miRNA and 19 lncRNA were acquired and a network of mRNA-miRNA-lncRNA was constructed, with some relationships like TBX5-hsa-mir-1270-TMPO-AS1 etc in the network (**Figure 6E**). Finally, pRRophetic predictions indicate that high TBX5 expression correlates with lower predicted IC_50_ values for drugs such as AG.014699 (p.adjust<0.05), suggesting tumours with elevated TBX5 expression may exhibit greater sensitivity to these therapeutic agents (**Figure 6F**). However, these computational predictions require further validation through subsequent experimental studies.

### 3.6 Molecular docking of GQD

**Figure 7:**
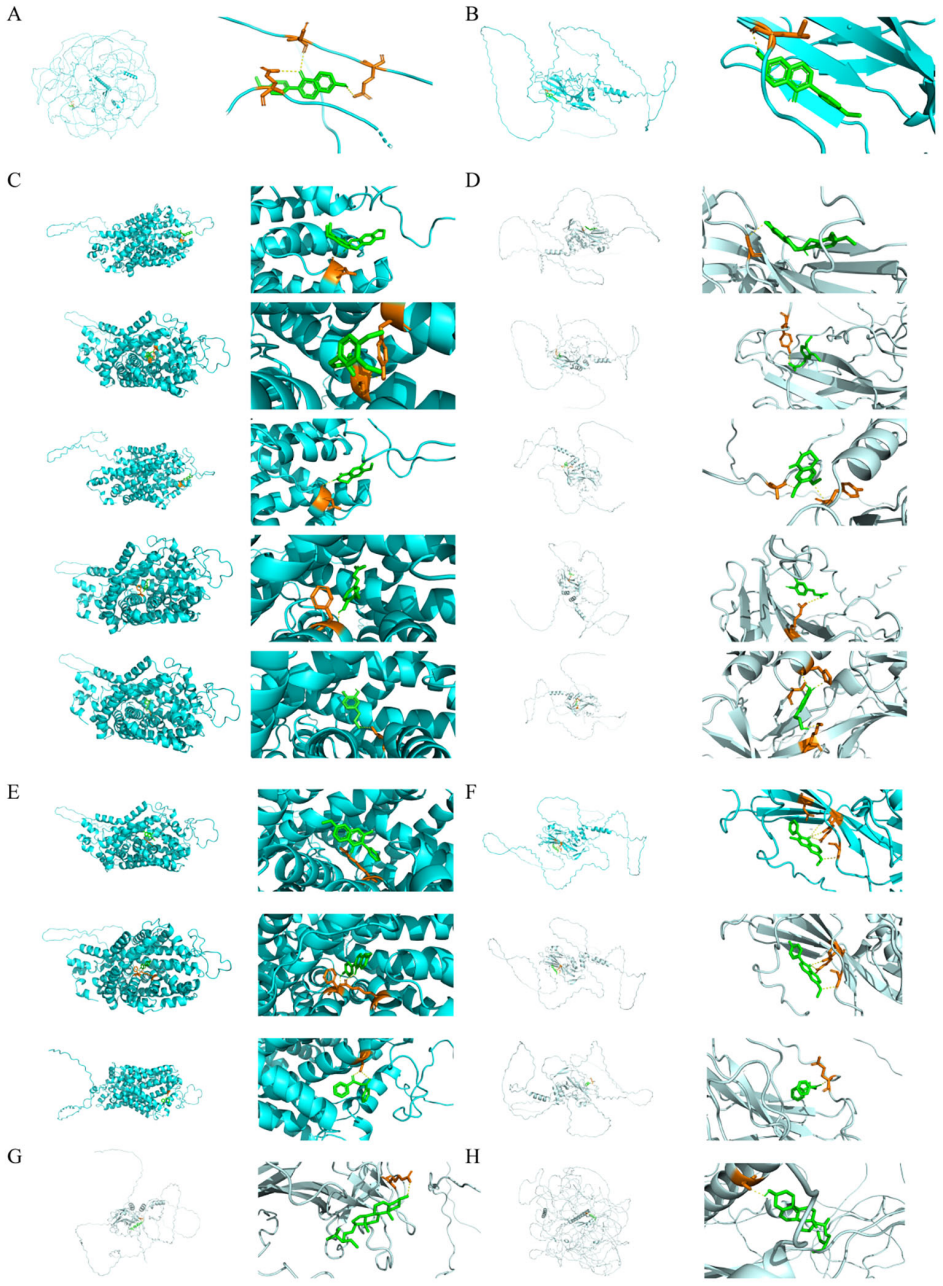
Molecular docking of GQD. **(A)** Docking results for MAP2 and formononetin. **(B)** Docking results for TBX5 and formononetin. **(C)** Docking results for SLC6A3 and the active component of Coptis chinensis. **(D)** Docking results for TBX5 with the active component of Coptis chinensis. **(E)** Docking results for SLC6A3 with the active component of Glycyrrhiza uralensis. **(F)**Docking results for TBX5 with the active component of Glycyrrhiza uralensis. **(G)** Docking results for MAP2 with the active component of Scutellaria baicalensis. **(H)** Docking results for TBX5 with the active component of Scutellaria baicalensis.

A total of four drugs were screened, including Pueraria lobata, Coptis chinensis, Glycyrrhiza uralensis, and Scutellaria baicalensis. Pueraria lobata obtained 14 active ingredients corresponding to 220 protein targets, converted into 176 corresponding genes. According to the results of spearman analysis in Pueraria lobata, the correlation between MAP2 and TBX5 was the highest and significant (cor = 0.288, pvalue = 1.214098e-13). After screening, formononetin was found to have a regulatory relationship with MAP2 (docking binding energy = -6.1 kcal/mol) (**Figure 7A**). TBX5 docked better with formononetin (docking binding energy = -6.5 kcal/mol) (**Figure 7B**). Coptis chinensis obtained 35 active ingredients corresponding to 227 protein targets, converted into 177 corresponding genes. The highest and significant correlation between SLC6A3 and TBX5 was found in Rhizoma Coptidis (cor = 0.271, pvalue = 3.284383e-12). 3,4-dimethoxy-(8CI), hydroxytyrosol, (R)-Canadine, Corydaldine and FER had regulatory relationship with SLC6A3 and TBX5 (docking binding energy < -5 kcal/mol) (**Figures 7C, D**). Glycyrrhiza uralensis obtained 165 active ingredients corresponding to 316 protein targets, converted into 246 corresponding genes, SLC6A3 showed the highest and significant correlation with TBX5 (cor = 0.271, pvalue = 3.284383e-12). And HMO, 7-Methoxy-2-methyl isoflavone, Karenzu etc had regulatory relationship with SLC6A3 and TBX5 (docking binding energy < -5 kcal/mol) (**Figures 7E, F**). *Scutellaria baicalensis* obtained 105 active ingredients corresponding to 233 protein targets, converted into 171 corresponding genes, MAP2 showed the highest and significant correlation with TBX5 (cor = 0.28, pvalue = 1.214098e-13). And beta-sitosterol had regulatory relationship with MAP2 and TBX5 (docking binding energy < -5 kcal/mol) (**Figures 7G, H**). Mesh parameters for the molecular docking process are detailed in the **Supplementary Table S8**. In silico molecular docking predicted that several active components from GQD, such as formononetin, might interact with TBX5. This provides a hypothetical molecular basis for the observed effects and warrants further investigation to validate these interactions.

### 3.7 Efficient knockdown of TBX5 gene expression using lentivirus-mediated shRNA in HCT116 cells

**Figure 8:**
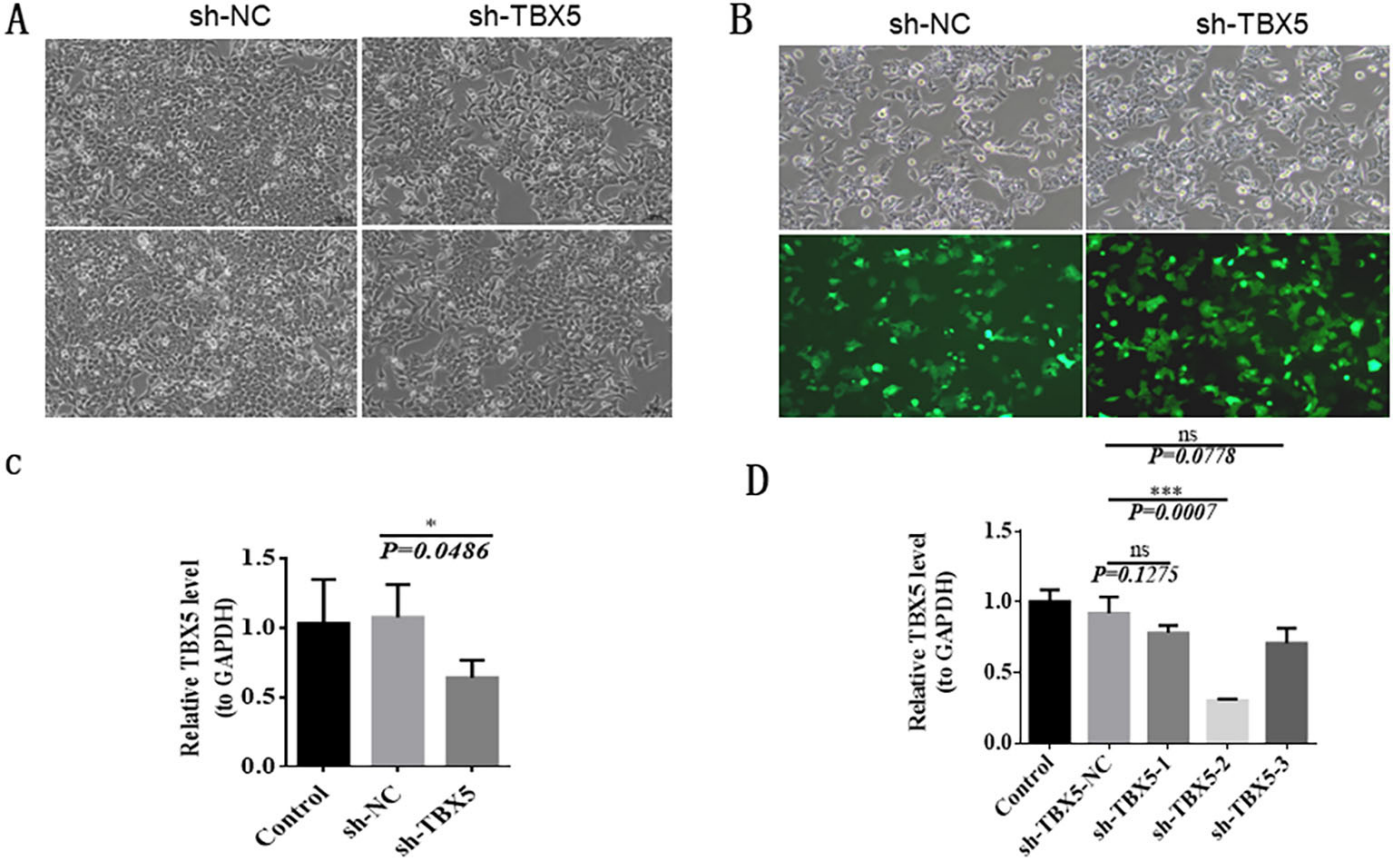
Efficient knockdown of TBX5 gene expression using lentivirus-mediated shRNA in HCT116 Cells. **(A)** The culture of HCT116 cells. **(B)** SH-TBX5 lentivirus transfected in 293T cells. **(C)** RT-qPCR validation to evaluate the efficiency of gene TBX5 silencing. **(D)** RT-qPCR validation of stable cell lines. (.*p*-value ≤ 0.1; *p-value ≤ 0.05; **p-value ≤ 0.01; ***p-value ≤ 0.001; TBX5 gene symbol is TBX5).

HCT116 cells were cultured and observed under the microscope. The cells were well attached to the culture dish, indicating that the cell growth status was good and suitable for further experiments (**Figure 8A**). sh-TBX5 lentivirus was successfully packaged in 293T cells with 100% green fluorescence intensity (**Figure 8B**). In order to determine the most effective knockdown vector, we performed RT-qPCR to evaluate the gene silencing efficiency. Compared with the sh-NC group, the expression level of TBX5 gene was significantly reduced in the sh-TBX5 group (**Figure 8C**). The successful establishment of a stable TBX5 knockdown cell line was confirmed and can be used for further analysis. To screen for stable cell lines, further RT-qPCR validation was performed. The results showed that compared with the sh-NC group, TBX5 gene expression was significantly decreased in the sh-TBX5-1, sh-TBX5–2 and sh-TBX5–3 groups, with the most obvious decrease in the sh-TBX5–2 group (**Figure 8D**). Therefore, the sh-TBX5–2 vector was selected for subsequent experiments.

### 3.8 GQD could inhibit tumor growth and TBX5 affected the development of CRC

**Figure 9:**
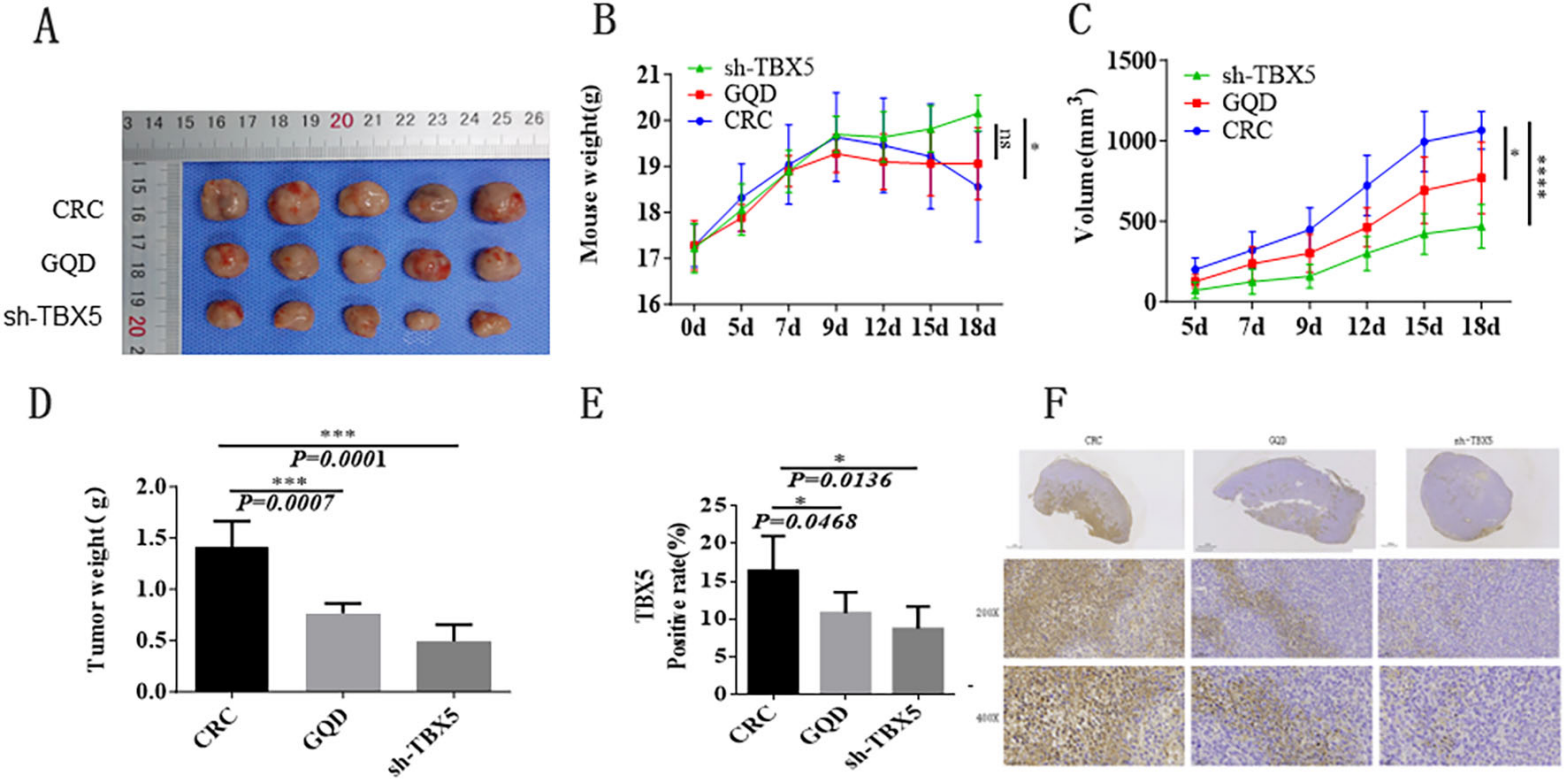
GQD could inhibit tumor growth and TBX5 affected the development of CRC. **(A)** The size of tumor among the control group, the GQD group and TBX5 lentivirus transfected group. **(B)** The weight of mice among the control group, the GQD group and TBX5 lentivirus transfected group. **(C)** The volume of tumor among the control group, the GQD group and TBX5 lentivirus transfected group. **(D)** The weight of tumor among the control group, the GQD group and TBX5 lentivirus transfected group. **(E)** Different TBX5 expressions among the control group, the GQD group and TBX5 lentivirus transfected group. **(F)** Immunohistochemistry results among the control group, the GQD group and TBX5 lentivirus transfected group. (.*p*-value ≤ 0.1; *p-value ≤ 0.05; **p-value ≤ 0.01; ***p-value ≤ 0.001; TBX5 gene symbol is TBX5).

The results of animal experiments showed that compared with the control group, the body weight of GQD group and TBX5 lentivirus transfected group increased and changed with time, and the tumors of the model group increased gradually, and the tumors of the GQD group and TBX5 lentivirus transfected group grew slowly (**Figure 9A-D**). Immunohistochemistry results showed that the GQD group showed low expression compared with the CRC group, and the sh-TBX5 group showed low expression compared with the CRC group. It indicates that GQD could inhibit tumor growth and TBX5 affected the development of CRC (**Figure 9E**).

## 4 Discussion

Colorectal cancer (CRC) is a prevalent malignant neoplasm affecting the gastrointestinal tract. In 2020, global statistics reported approximately 1.9 million new cases and 900,000 deaths from CRC. It ranks second in mortality rates among cancers, contributing to 9.4% of cancer-related deaths (28, 29). Treatment options for CRC have expanded over time to include surgical resection, radiotherapy, immunotherapy, palliative chemotherapy, targeted therapy, and local ablation of metastatic lesions. Despite advancements in CRC treatment, the rising incidence and demographic shift towards younger populations present significant public health challenges (28).Recent research has identified TBX5 as a novel tumor-regulating gene implicated in esophageal adenocarcinoma and colon cancer (30, 31). Studies by Palles et al. (32) revealed an association between TBX5 (rs2701108) and the susceptibility to Barrett esophagus and esophageal adenocarcinoma through genome-wide association studies. Furthermore, genes encoding transcription factors for Barrett esophagus have implications in the development of the chest, diaphragm, and esophagus. Becker et al. (30) corroborated the involvement of rs2701108 in Barrett esophagus and esophageal adenocarcinoma development. TBX5 is speculated to play a crucial role in diaphragm development, and mutations in the TBX5 gene may lead to esophageal hiatal hernia and gastroesophageal reflux disease, contributing to the progression from Barrett esophagus to esophageal adenocarcinoma. Moreover, the β-catenin-TBX5 complex, particularly involving Yes-associated protein 1 (YAP1), plays a significant role in β-catenin-driven cancer, notably in colon cancer (31). Therefore, our study utilized various bioinformatics tools to investigate the biological function of TBX5 in CRC and explored the interaction between GQD and TBX5 through molecular docking. Our findings demonstrated that GQD influences CRC development by modulating TBX5, highlighting TBX5 as a transcription factor with nuclear transcriptional regulatory functions upon activation. Notably, TBX5 expression is upregulated in the nucleus of CRC, particularly in adenocarcinomas across different tumor stages. Furthermore, our experimental data revealed a significant correlation between TBX5 expression levels and patient prognosis, indicating a worse prognosis with higher gene expression levels. These results suggest a positive association between TBX5 levels and colorectal cancer progression, contrasting its role in lung cancer regulation.

This study found that in colorectal cancer (CRC) patients with high TBX5 expression, the infiltration levels of myeloid-derived suppressor cells (MDSCs) and regulatory T cells (Tregs) in the tumor microenvironment (TME) are significantly elevated. Notably, high MDSC infiltration is strongly associated with decreased overall survival of patients, suggesting that TBX5 may promote immune escape in CRC by regulating the infiltration of immunosuppressive cells. As key immunosuppressive cells in the TME, recent studies have demonstrated that the immunosuppressive activity of MDSCs depends on the regulation of cholesterol metabolism. For instance, RIPK3 deficiency can induce cholesterol metabolic disorders in MDSCs, thereby enhancing their immunosuppressive capacity and promoting colorectal cancer progression (33, 34). Additionally, gasdermin D deficiency facilitates the differentiation of granulocytic MDSCs by reducing mitochondrial DNA release, further exacerbating tumor immune escape (35). As a transcription factor, TBX5 may influence the recruitment and functional activation of MDSCs in the TME by transcriptionally regulating MDSC-related chemokines (e.g., CXCL1, CSF1) or metabolism-associated enzymes. However, the specific regulatory mechanism of TBX5 on MDSCs requires further validation through functional experiments, and systematic analysis of its downstream target genes is necessary to fully elucidate the critical role of TBX5 in remodeling the immune microenvironment of colorectal cancer.

GO and KEGG enrichment analysis results based on TBX5 indicate that high TBX5 expression is closely associated with key oncogenic pathways, including extracellular matrix (ECM)-receptor interaction, epithelial-mesenchymal transition (EMT), cancer stemness, and angiogenesis. This finding is consistent with the conclusion of this study that high TBX5 expression correlates with poor prognosis in patients. Multiple studies have pointed out that epithelial-mesenchymal transition can promote the progression of colorectal cancer (36, 37). Notably, previous studies have shown that the function of TBX5 is to activate the expression of EMT-related markers (e.g., Wnt2 and Fgf10) and their downstream target genes (e.g., Bmp4 and Spry2) (37). Therefore, we speculate that in CRC, high TBX5 expression is likely to continuously activate this conserved EMT program, thereby enhancing the invasion, metastasis, and cancer stem cell properties of tumor cells, and ultimately promoting disease progression (16).

Radix Astragali in Gegen Qinlian decoction exhibits properties of replenishing qi, solidifying the surface, detoxifying, and promoting suppuration. Scutellaria baicalensis possesses heat-clearing, drying, hemostatic, and detoxifying capabilities. Pueraria is known for invigorating body fluids, relieving thirst, and reducing heat. Licorice serves as an agent for adjustment, heat-clearing, and detoxification, with all these ingredients commonly utilized to enhance spleen function, alleviate diarrhea, clear heat, remove dampness, promote hemostasis, and detoxify. Recent studies have revealed the complex chemical composition of this compound, with modern pharmacological investigations demonstrating the efficacy of Gegen Qinlian decoction and its active compounds in inflammation inhibition, antibacterial action, antioxidation, and intestinal mucosa protection. Research has indicated that Gegen Qinlian decoction can potentiate the impact of PD-1 in blocking microsatellite stability in colorectal cancer by reshaping the intestinal microbiota and tumor microenvironment (38–41). Widely employed in colorectal cancer treatment, Gegen Qinlian decoction contains various active constituents (such as baicalin, licorice flavonoids, berberine) that significantly reduce inflammatory factor levels, modulate oxidative stress, and exhibit anti-inflammatory and anti-infective effects (41, 42). The decoction can downregulate protein kinase B activity, inhibit its phosphorylation to induce tumor cell apoptosis, and exert anticancer effects by suppressing the transcriptional process of the phosphatidylinositol 3-kinase-3C gene. Formononetin and SLC6A3 regulate TBX5 expression, facilitating tumor cell apoptosis (40). This provides a novel direction for further elucidating the molecular mechanisms underlying the compound’s promotion of colorectal cancer cell apoptosis and exertion of antitumor effects.

In this study, we investigated the expression of TBX5 in colorectal cancer, its prognostic implications, its association with immunotherapy and drug sensitivity, and established a regulatory network for molecular docking. Our findings identified TBX5 as a oncogene in colon cancer. Moreover, we observed a synergistic effect between Gegen Qinlian decoction and TBX5 in regulating apoptosis in primary colorectal cancer, suggesting potential implications for patient prognosis, immunotherapy, and drug sensitivity. However, this study has several limitations. Firstly, the pan-cancer analysis was not subjected to multiple test correction, nor was tumor purity incorporated into the model as a covariate. This may increase the probability of false positives and potentially affect the accuracy of evaluating the correlation between genes and tumors due to the neglect of non-tumor cell interference. In the future, it will be necessary to introduce FDR correction and integrate tumor purity estimation algorithms to improve the rigor of the analysis. Secondly, the validation of gene knockout efficiency was only performed at the transcript level via qPCR. Although the results are highly consistent with the observed phenotypes, future validation at the protein level via Western blot will further solidify the conclusions. Additionally, the current work mainly focuses on preliminary exploration at the theoretical and molecular levels, and the specific underlying mechanisms require further elucidation through more in-depth experimental and clinical studies. On this basis, we will focus on in-depth investigation of the function of TBX5 in colorectal cancer as a future research direction.

**Figure 10:**
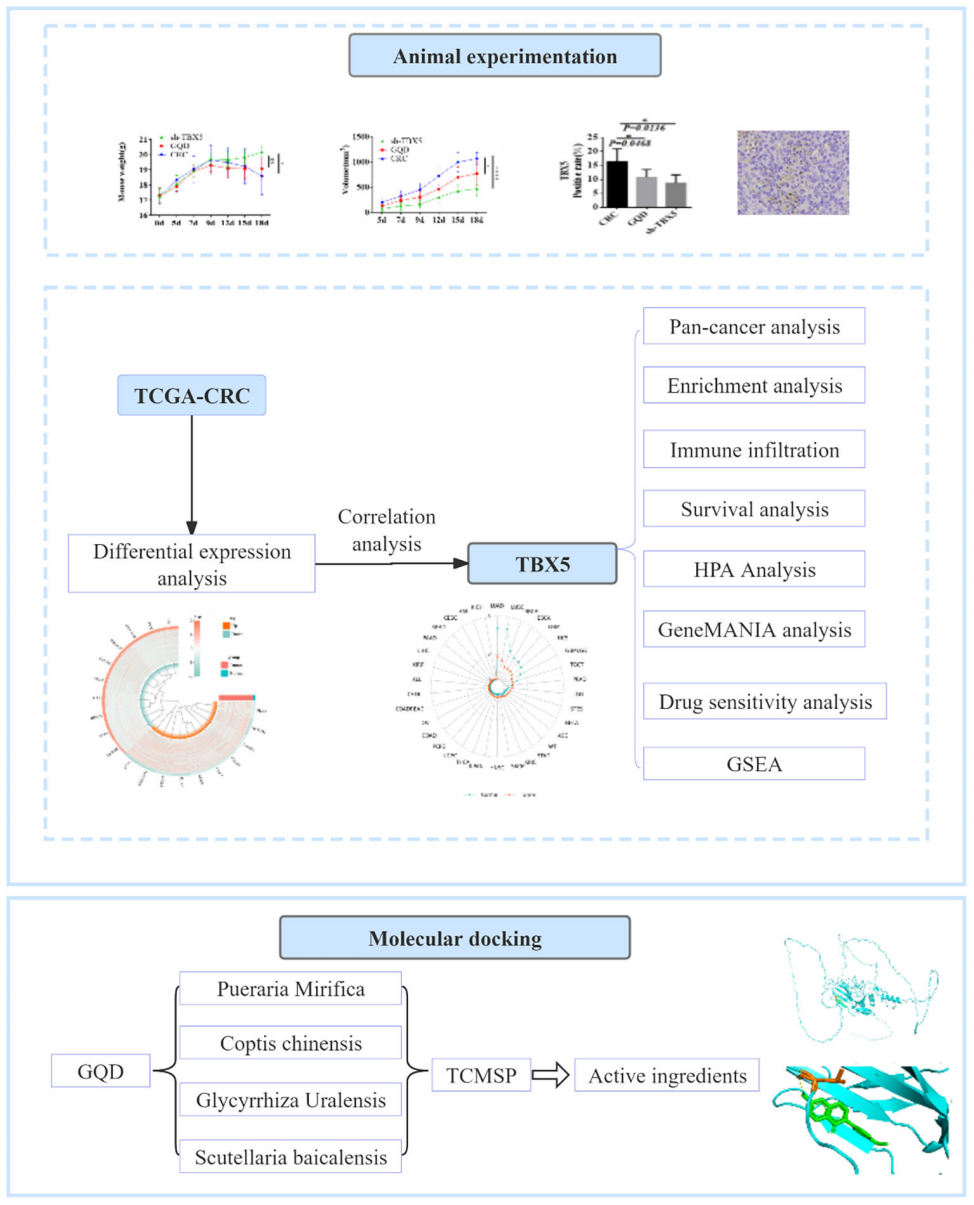
Analysis explanation with a detailed flow chart of this study.

In this study, we examined the mRNA expression patterns of TBX5, its prognostic significance, its correlation with tumor-infiltrating immune cells, and related pathways across different cancer types using a multi-omic bioinformatics approach (**Figure 10**). Our findings confirmed that elevated TBX5 expression is linked to unfavorable prognosis and an immunosuppressive tumor microenvironment in colorectal cancer (CRC) patients. These outcomes underscore the relevance of TBX5 expression in predicting cancer prognosis and therapy outcomes, highlighting key avenues for future investigation and validation.

## Data Availability

The original contributions presented in the study are included in the article/**Supplementary Material**. Further inquiries can be directed to the corresponding author.

## References

[1] Keum N Giovannucci E . Global burden of colorectal cancer: emerging trends, risk factors and prevention strategies. Nat Rev Gastroenterol Hepatol. (2019) 16:713–32. doi: 10.1038/s41575-019-0189-8, PMID: 31455888

[2] Sung H Ferlay J Siegel RL Laversanne M Soerjomataram I Jemal A . Global cancer statistics 2020: GLOBOCAN estimates of incidence and mortality worldwide for 36 cancers in 185 countries. CA Cancer J Clin. (2021) 71:209–49. doi: 10.3322/caac.21660, PMID: 33538338

[3] Chen X Jansen L Guo F Hoffmeister M Chang-Claude J Brenner H . Smoking, genetic predisposition, and colorectal cancer risk. Clin Transl Gastroenterol. (2021) 12:e00317. doi: 10.14309/ctg.0000000000000317, PMID: 33646204 PMC7925134

[4] Latino-Martel P Srour B Ginhac J Bigey J Ancellin R . During and after cancer: identification of high-risk nutritional situations. Rev Prat. (2021) 71:155–59. doi: 10.1016/j.pratic.2020.11.021, PMID: 34160971

[5] Dong Y Zhou J Zhu Y Luo L He T Hu H . Abdominal obesity and colorectal cancer risk: systematic review and meta-analysis of prospective studies. Biosci Rep. (2017) 37(6):BSR20170945. doi: 10.1042/bsr20170945, PMID: 29026008 PMC5725611

[6] Vieira AR Abar L Chan DSM Vingeliene S Polemiti E Stevens C . Foods and beverages and colorectal cancer risk: a systematic review and meta-analysis of cohort studies, an update of the evidence of the WCRF-AICR Continuous Update Project. Ann Oncol. (2017) 28:1788–802. doi: 10.1093/annonc/mdx171, PMID: 28407090

[7] Gao R Gao Z Huang L Qin H . Gut microbiota and colorectal cancer. Eur J Clin Microbiol Infect Dis. (2017) 36:757–69. doi: 10.1007/s10096-016-2881-8, PMID: 28063002 PMC5395603

[8] Lynch SV Pedersen O . The human intestinal microbiome in health and disease. N Engl J Med. (2016) 375:2369–79. doi: 10.1056/NEJMra1600266, PMID: 27974040

[9] Allemani C Matsuda T Di Carlo V Harewood R Matz M Nikšić M . Global surveillance of trends in cancer survival 2000-14 (CONCORD-3): analysis of individual records for 37 513 025 patients diagnosed with one of 18 cancers from 322 population-based registries in 71 countries. Lancet. (2018) 391:1023–75. doi: 10.1016/s0140-6736(17)33326-3, PMID: 29395269 PMC5879496

[10] Naiche LA Harrelson Z Kelly RG Papaioannou VE . T-box genes in vertebrate development. Annu Rev Genet. (2005) 39:219–39. doi: 10.1146/annurev.genet.39.073003.105925, PMID: 16285859

[11] Papaioannou VE . The T-box gene family: emerging roles in development, stem cells and cancer. Development. (2014) 141:3819–33. doi: 10.1242/dev.104471, PMID: 25294936 PMC4197708

[12] Khalil AA Sivakumar S Lucas FAS McDowell T Lang W Tabata K . TBX2 subfamily suppression in lung cancer pathogenesis: a high-potential marker for early detection. Oncotarget. (2017) 8:68230–41. doi: 10.18632/oncotarget.19938, PMID: 28978111 PMC5620251

[13] Arora R Metzger RJ Papaioannou VE . Multiple roles and interactions of Tbx4 and Tbx5 in development of the respiratory system. PloS Genet. (2012) 8:e1002866. doi: 10.1371/journal.pgen.1002866, PMID: 22876201 PMC3410851

[14] Mori AD Bruneau BG . TBX5 mutations and congenital heart disease: Holt-Oram syndrome revealed. Curr Opin Cardiol. (2004) 19:211–5. doi: 10.1097/00001573-200405000-00004, PMID: 15096952

[15] He ML Chen Y Peng Y Jin D Du D Wu J . Induction of apoptosis and inhibition of cell growth by developmental regulator hTBX5. Biochem Biophys Res Commun. (2002) 297:185–92. doi: 10.1016/s0006-291x(02)02142-3, PMID: 12237100

[16] Yu J Ma X Cheung KF Li X Tian L Wang S . Epigenetic inactivation of T-box transcription factor 5, a novel tumor suppressor gene, is associated with colon cancer. Oncogene. (2010) 29:6464–74. doi: 10.1038/onc.2010.370, PMID: 20802524

[17] Lu JZ Ye D Ma BL . Constituents, pharmacokinetics, and pharmacology of gegen-qinlian decoction. Front Pharmacol. (2021) 12:668418. doi: 10.3389/fphar.2021.668418, PMID: 34025427 PMC8139575

[18] Lv J Jia Y Li J Kuai W Li Y Guo F . Gegen Qinlian decoction enhances the effect of PD-1 blockade in colorectal cancer with microsatellite stability by remodelling the gut microbiota and the tumour microenvironment. Cell Death Dis. (2019) 10:415. doi: 10.1038/s41419-019-1638-6, PMID: 31138779 PMC6538740

[19] Love MI Huber W Anders S . Moderated estimation of fold change and dispersion for RNA-seq data with DESeq2. Genome Biol. (2014) 15:550. doi: 10.1186/s13059-014-0550-8, PMID: 25516281 PMC4302049

[20] Lei J Qu T Cha L Tian L Qiu F Guo W . Clinicopathological characteristics of pheochromocytoma/paraganglioma and screening of prognostic markers. J Surg Oncol. (2023) 128:510–18. doi: 10.1002/jso.27358, PMID: 37272486

[21] Maffeo D Rina A Serio VB Markou A Powrózek T Constâncio V . The evidence base for circulating tumor DNA-methylation in non-small cell lung cancer: A systematic review and meta-analysis. Cancers (Basel). (2024) 16(21):3641. doi: 10.3390/cancers16213641, PMID: 39518079 PMC11544801

[22] Yan J Liu Y Liu T Zhu Q . A predictive and prognostic model for metastasis risk and prognostic factors in gastrointestinal signet ring cell carcinoma. Eur J Med Res. (2024) 29:545. doi: 10.1186/s40001-024-02135-5, PMID: 39538294 PMC11562313

[23] Heagerty PJ Lumley T Pepe MS . Time-dependent ROC curves for censored survival data and a diagnostic marker. Biometrics. (2000) 56:337–44. doi: 10.1111/j.0006-341x.2000.00337.x, PMID: 10877287

[24] Hänzelmann S Castelo R Guinney J . GSVA: gene set variation analysis for microarray and RNA-seq data. BMC Bioinf. (2013) 14:7. doi: 10.1186/1471-2105-14-7, PMID: 23323831 PMC3618321

[25] Yu G Wang LG Han Y He QY . clusterProfiler: an R package for comparing biological themes among gene clusters. Omics. (2012) 16:284–7. doi: 10.1089/omi.2011.0118, PMID: 22455463 PMC3339379

[26] Charoentong P Finotello F Angelova M Mayer C Efremova M Rieder D . Pan-cancer immunogenomic analyses reveal genotype-immunophenotype relationships and predictors of response to checkpoint blockade. Cell Rep. (2017) 18:248–62. doi: 10.1016/j.celrep.2016.12.019, PMID: 28052254

[27] Geeleher P Cox N Huang RS . pRRophetic: an R package for prediction of clinical chemotherapeutic response from tumor gene expression levels. PloS One. (2014) 9:e107468. doi: 10.1371/journal.pone.0107468, PMID: 25229481 PMC4167990

[28] Xi Y Xu P . Global colorectal cancer burden in 2020 and projections to 2040. Transl Oncol. (2021) 14:101174. doi: 10.1016/j.tranon.2021.101174, PMID: 34243011 PMC8273208

[29] Yang M Sun M Zhang H . The interaction between epigenetic changes, EMT, and exosomes in predicting metastasis of colorectal cancers (CRC). Front Oncol. (2022) 12:879848. doi: 10.3389/fonc.2022.879848, PMID: 35712512 PMC9197117

[30] Becker J May A Gerges C Anders M Schmidt C Veits L . The Barrett-associated variants at GDF7 and TBX5 also increase esophageal adenocarcinoma risk. Cancer Med. (2016) 5:888–91. doi: 10.1002/cam4.641, PMID: 26783083 PMC4864818

[31] Rosenbluh J Nijhawan D Cox AG Li X Neal JT Schafer EJ . β-Catenin-driven cancers require a YAP1 transcriptional complex for survival and tumorigenesis. Cell. (2012) 151:1457–73. doi: 10.1016/j.cell.2012.11.026, PMID: 23245941 PMC3530160

[32] Palles C Chegwidden L Li X Findlay JM Farnham G Castro Giner F . Polymorphisms near TBX5 and GDF7 are associated with increased risk for Barrett’s esophagus. Gastroenterology. (2015) 148:367–78. doi: 10.1053/j.gastro.2014.10.041, PMID: 25447851 PMC4315134

[33] Zhou D Chen Y Liu X He J Shen L He Y . Cholesterol 25-hydroxylase enhances myeloid-derived suppressor cell (MDSC) immunosuppression via the stimulator of interferon genes (STING)-tank-binding kinase 1 (TBK1)-receptor-interacting protein kinase 3 (RIPK3) pathway in colorectal cancer. MedComm. (2020) 2025) 6:e70411. doi: 10.1002/mco2.70411, PMID: 41020041 PMC12475974

[34] Chen Y Xu Y Zhao H Zhou Y Zhang J Lei J . Myeloid-derived suppressor cells deficient in cholesterol biosynthesis promote tumor immune evasion. Cancer Lett. (2023) 564:216208. doi: 10.1016/j.canlet.2023.216208, PMID: 37150500

[35] Gu M Chen W Ding S Lin Z Qian L Xiao W . Deletion of gasdermin D promotes granulocytic myeloid-derived suppressor cell differentiation by decreased release of mitochondrial DNA to promote tumor escape. Cancer Immunol Immunother. (2025) 74:277. doi: 10.1007/s00262-025-04104-1, PMID: 40711527 PMC12297055

[36] Huang H Wang L Gao S Wang H . COMP promotes the progression of colorectal cancer by regulating epithelial mesenchymal transition. BMC Cancer. (2025) 25:1710. doi: 10.1186/s12885-025-15000-3, PMID: 41194017 PMC12587721

[37] Zhang Y Li Y Zuo Z Li T An Y Zhang W . An epithelial-mesenchymal transition-related mRNA signature associated with the prognosis, immune infiltration and therapeutic response of colon adenocarcinoma. Pathol Oncol Res. (2023) 29:1611016. doi: 10.3389/pore.2023.1611016, PMID: 36910014 PMC9998511

[38] Wei M Li H Li Q Qiao Y Ma Q Xie R . Based on network pharmacology to explore the molecular targets and mechanisms of gegen qinlian decoction for the treatment of ulcerative colitis. BioMed Res Int. (2020) 2020:5217405. doi: 10.1155/2020/5217405, PMID: 33299870 PMC7710413

[39] Liu SY Hu LL Wang SJ Liao ZL . Administration of modified Gegen Qinlian decoction for hemorrhagic chronic radiation proctitis: A case report and review of literature. World J Clin cases. (2023) 11:1129–36. doi: 10.12998/wjcc.v11.i5.1129, PMID: 36874424 PMC9979297

[40] Fan Q Guo L Guan J Chen J Fan Y Chen Z . Network pharmacology-based study on the mechanism of gegen qinlian decoction against colorectal cancer. Evid Based Complement Alternat Med. (2020) 2020:8897879. doi: 10.1155/2020/8897879, PMID: 33294000 PMC7714584

[41] Lin X Xu L Tan H Zhang X Shao H Yao L . The potential effects and mechanisms of Gegen Qinlian Decoction in oxaliplatin-resistant colorectal cancer based on network pharmacology. Heliyon. (2022) 8:e11305. doi: 10.1016/j.heliyon.2022.e11305, PMID: 36353164 PMC9638763

[42] Li Y Li ZX Xie CY Fan J Lv J Xu XJ . Gegen Qinlian decoction enhances immunity and protects intestinal barrier function in colorectal cancer patients via gut microbiota. World J Gastroenterol. (2020) 26:7633–51. doi: 10.3748/wjg.v26.i48.7633, PMID: 33505141 PMC7789057

