# Supplementary material for: Study on the regulation mechanism of TBX5 gene and Gegen Qinlian decoction on colorectal cancer
Source: Front Oncol. 2026 Jan 14;15:1732015. doi: 10.3389/fonc.2025.1732015 (PMC12847017; doi:10.3389/fonc.2025.1732015)
Supplement: Supplementary file 1 [file DataSheet1.zip › Supplementary Table 1.docx]

Supplementary Table S1 Preparation of plasmid 1 and transfection reagent dilution solution 2

| Centrifuge tube 1 (plasmid DNA) | Centrifuge tubes 2 (transfection reagents) |
| --- | --- |
| Lentiviral vector 5 μg | PEI transfection reagent 20 μl |
| pH1 vector 3.75μg |  |
| pH2 vector 1.25 μg |  |
| DMEM serum-free medium 1000 μl | DMEM serum-free medium 480 μl |
