# Supplementary material for: Study on the regulation mechanism of TBX5 gene and Gegen Qinlian decoction on colorectal cancer
Source: Front Oncol. 2026 Jan 14;15:1732015. doi: 10.3389/fonc.2025.1732015 (PMC12847017; doi:10.3389/fonc.2025.1732015)
Supplement: Supplementary file 1 [file DataSheet1.zip › Supplementary Table 2.docx]

Supplementary Table S2 Primer sequence list

| primer | sequences（5‘-3’） |
| --- | --- |
| TBX5 F | AAGACCTGGCCTAAAGAGGT |
| TBX5 R | CCTCCATGCCCTCCAACTA |
| internal reference H-GAPDH F | CCCATCACCATCTTCCAGG |
| internal reference H-GAPDH R | CATCACGCCACAGTTTCCC |
